# Effectiveness of the Support From Community Health Workers and Health Care Professionals on the Sustained Use of Wearable Monitoring Devices Among Community-Dwelling Older Adults: Feasibility Randomized Controlled Trial

**DOI:** 10.2196/52435

**Published:** 2024-11-18

**Authors:** Arkers Kwan Ching Wong, Jonathan Bayuo, Jing Jing Su, Frances Kam Yuet Wong, Karen Kit Sum Chow, Bonnie Po Wong, Siu Man Wong, Vivian Hui

**Affiliations:** 1 School of Nursing Hong Kong Polytechnic University Hong Kong China (Hong Kong); 2 Hong Kong Lutheran Social Service Homantin China (Hong Kong)

**Keywords:** wearable monitoring device, lay worker, smartwatch, older adult, nurse, engagement, attrition, engagement, wearable, user experience

## Abstract

**Background:**

The wearable monitoring device (WMD) is emerging as a promising tool for community-dwelling older adults to monitor personal health, enhance awareness of their activities, and promote healthy behaviors. However, the sustained use of WMDs among this population remains a significant challenge.

**Objective:**

This study aims to implement an interventional program that promotes and motivates the continued use of WMDs among older adults through a peer and professional support approach. This program will facilitate the integration of WMDs into their daily lives.

**Methods:**

This feasibility trial examined the following: (1) the usability of the WMD from the users’ perspectives; (2) the feasibility of the Live With Wearable Monitoring Device program; and (3) the effectiveness of the Live With Wearable Monitoring Device program among community-dwelling older adults. The intervention, based on Self-Determination Theory, involved using the Live With Wearable Monitoring Device program over a 3-month period, with ongoing professional and peer support provided by community health workers, aided by a nurse and social workers. This support included 1 home visit and biweekly communication via WhatsApp. Data were collected at baseline and at 1, 3, and 6 months.

**Results:**

A total of 39 participants were enrolled in the intervention group, while 37 participants were in the control group. The recruitment rate was high (76/89, 85%), and the attrition rate was low (8/76, 11%), indicating that the program is feasible for older adults. Participants in the intervention group exhibited higher self-efficacy, lower anxiety levels, and used the smartwatch more frequently, in terms of both days and hours, compared with the control group. A between-group difference was observed in self-efficacy between the intervention and control groups (β=3.31, 95% CI 0.36-6.25, *P*=.03), with statistically significant higher mean values recorded at all 4 time points.

**Conclusions:**

It is clear that merely providing a WMD to older adults does not guarantee its usage, particularly for those unfamiliar with how to utilize its health-related functions in their daily routines. This study implemented a theory-based program aimed at enhancing the ongoing use of WMDs among older adults, suggesting that continuous professional and peer support may significantly influence WMD usage.

**Trial Registration:**

ClinicalTrials.gov NCT05269303; https://clinicaltrials.gov/ct2/show/NCT05269303

## Introduction

### Background

Given the ever-growing aging population, strategies such as health education and self-care management to promote older adults’ health are becoming increasingly important [1]. With digital technology, older adults can engage more actively in these strategies, supporting the prevention, early detection, and management of chronic diseases [1,2]. Wearable monitoring devices (WMDs) are among the most popular electronic tools today for encouraging older adults to select or adjust suitable types and frequencies of health-promoting activities based on real-time personal health data. By becoming more active in their own care, older adults can improve their physical fitness and work toward their goal of remaining in their community for as long as possible [2].

A WMD is defined as body-worn technology that enables continuous monitoring of individual health-related activities without restricting or interrupting movement [2]. Although a wide range of WMDs are available on the market, these devices generally allow users to only capture key personal health indicators, such as blood pressure, pulse, physical activity levels, posture, and body weight. Data collected by commercially available WMDs are typically transmitted to a cloud platform or the manufacturer’s server for processing. Users then receive automated, general feedback or alerts based on these processed data, guiding them in taking further actions [3]. Some of the latest WMDs can even provide users with high-touch, personalized feedback when the server is directly connected to the smartphones of supporting professionals, such as doctors or nurses [4]. These supporting professionals use users’ real-time health parameters to provide meaningful, timely feedback and establish a communication channel, positively impacting users’ health and well-being [4]. Most prior research supports the view that WMDs enhance older adults’ sense of security and quality of life [5], reduce sedentary behaviors and health care service utilization [6], improve gait balance [1], and lead to reductions in blood pressure and body mass index [7]. Some studies also suggest that WMDs can assist both lay caregivers and health care professionals in tracking care, identifying issues promptly, and providing immediate support to help older family members or clients maintain their independence [8]. Despite the positive impact of WMDs, it is important to note that certain issues and limitations exist. Concerns about privacy and data sharing, limited battery capacity, and accuracy in screening and prediction highlight challenges regarding the reliability and validity of wearables as detection and prediction tools [9].

Given the potential benefits of WMDs in supporting health self-management for older adults, commercial studies report that WMD sales have risen sharply in recent years and are expected to continue growing annually over the next 5 years [10]. However, despite widespread adoption, reports indicate that older adults often discontinue use within a few months, with an abandonment rate exceeding 30% [11-13]. Another longitudinal study echoed this finding, showing that approximately 50% of new WMD users stop using the device within 2 weeks [14]. Possible explanations for this low adherence rate are issues with how the WMDs were implemented and challenges users face in integrating the device into their daily routines, despite its usefulness.

Often, the motivation to continue using WMDs depends on the extent to which users feel the device fulfills their needs, aligns with their goals, and meets their expectations [15,16]. However, while commercially available WMDs—such as those produced by Apple, Fitbit, and Garmin—include goal-setting features, they lack built-in guidance on maximizing the effectiveness of these features. They also offer limited support for action planning and for identifying facilitators and barriers to their use in daily activities. These issues can discourage individuals from using the device, even if they find it useful. Additionally, previous research has found that, beyond design, features, perceived usefulness, and ease of use, the extent to which the device empowers users as responsible for their own health decisions is a crucial factor motivating older adults to sustain its use [17]. As health care professionals can provide instant support whenever a WMD detects abnormal health findings, older adults may feel less compelled to take ownership of their health and engage in self-monitoring. This situation leaves little room for their involvement in planning their health management, which may hinder their determination and motivation to comply with WMD usage.

Regarding the implementation of WMDs, the typical approach has been to provide participants with the device, based on the assumption that they will use it because they understand the benefits [14]. The problem with this approach is that it is passive and does not consider that people may become demotivated to use the device over time. Therefore, more innovative strategies are needed to sustain usage. Given that challenges are likely to arise during device use, an active approach that offers ongoing professional or peer support may be beneficial. Although in a different context, a recent study on the sustained use of an mHealth app found that ongoing follow-up support was effective in equipping community-dwelling older adults with the skills needed to navigate the app while addressing technical issues and limited social support [18]. This suggests that the availability of ongoing professional and peer support provides a platform for older adults to resolve the issues they encounter, thereby potentially helping to sustain their interest in the app.

The World Health Organization’s global strategy for digital health emphasizes the importance of empowering users to integrate technology into their daily routines [19]. Community health workers (CHWs), who share similar backgrounds and come from the same community, are highly regarded as peers who can engage older adults in using electronic tools for self-care management [20]. Taylor and his colleagues [21] noted that CHWs possess high health literacy and strong interpersonal communication skills, enabling them to serve as role models for their peers and improve engagement in self-care and healthy behaviors. Previous programs involving CHWs in key support roles have been effective in improving health outcomes [22], reducing health care costs [23], decreasing the use of health care services [24], and enhancing the quality of care [25] among older adults. With the support of CHWs, older adults can familiarize themselves with the technical design and functionality of the devices, track and manage their health parameters, overcome barriers to using WMDs in their daily lives, codevelop action plans to achieve health goals, and receive personalized feedback on their performance. This support may ultimately facilitate and motivate them to continue using the devices.

Motivating older adults to sustain the use of WMDs is an emerging area of research, although it currently has a limited evidence base. Much of the existing research focuses on exploring the motivational factors that influence WMD use among older adults, the development process of these devices, and their effects on various populations [26]. However, there are only a few interventional studies aimed at empowering and engaging community-dwelling older adults in the sustainable use of WMDs in their daily lives. A recent 1-group pretest-posttest design study implemented a 3-month program that included weekly reminders and monthly phone calls from a research team member to promote the use of a Fitbit device among older adults with osteoarthritis. The results showed that 96% of participants remained in the study, and Fitbit wear time was high, although no health data were captured [27]. Another study provided a group of community-dwelling individuals with WMDs along with support services from a dietitian, demonstrating an improvement in their continued use of the devices [28]. Our study is the first to use a peer support approach to engage community-dwelling older adults in the continued use of WMDs in their daily routines. It aims to assess the usability of the WMD, the program’s feasibility, and its effectiveness from the perspective of older adults. If proven successful, the program could offer a solution to motivate older adults to consistently use WMDs for self-care management, thereby improving their health and quality of life in the long term.

### Conceptual Framework

This study was guided by Self-Determination Theory (SDT) [29], which posits that individuals are more likely to adhere to a particular behavior when they have intrinsic motivation to do so.

In this study, we aimed to sustain the intrinsic motivation of community-dwelling older adults to facilitate their continued use of WMDs in their daily routines. According to SDT, intrinsic motivation can be determined and enhanced by 3 factors: competence, autonomy, and relatedness.

**Figure 1:**
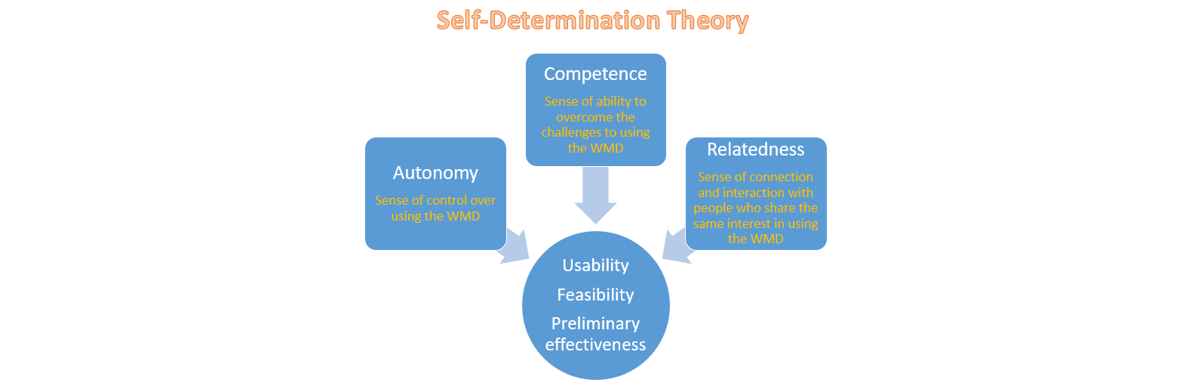
Conceptual framework. WMD: wearable monitoring device.

Competence refers to the sense of ability to overcome challenges associated with an activity or behavior. To facilitate the achievement of this component, the study used a home visit approach by the CHW and the nurse case manager to demonstrate the use of the WMD to participants. This 1-on-1 approach helped identify and resolve barriers inherent in the participants’ environments, equipping them with the necessary knowledge and skills for using the WMD effectively. Autonomy refers to a sense of control over an activity or behavior. Through a structured follow-up approach that included biweekly calls from the nurse case manager, emerging concerns regarding the use of the WMD were identified and addressed, facilitating independent use while still providing ongoing support as needed. Relatedness refers to a sense of connection and interaction with others who share an interest in an activity or behavior. To increase the adherence rate of community-dwelling older adults in using WMDs in their daily lives, the present program included components designed to teach them the knowledge and skills needed to effectively use the WMD and overcome technical difficulties. It also provided a clear rationale for suggestions and offered various options for incorporating WMDs into their routines. Additionally, the program aimed to strengthen interpersonal connections and support from family members and peers, facilitating the continued use of the WMD (Figure 1).

## Methods

### Study Design, Participants, and Recruitment

This study utilized a feasibility randomized controlled trial design. The study was conducted at 5 community centers affiliated with a local nongovernmental organization. Participants who were members and expressed interest in the program were screened and recruited based on the following criteria: (1) aged 60 or older, (2) owning a smartphone, (3) able to communicate in Cantonese or Mandarin, and (4) having internet access at home or elsewhere. Potential participants were excluded based on the following criteria: (1) a confirmed diagnosis of cognitive impairment, (2) being bedbound, (3) currently owning a WMD, and (4) having previously participated in other wearable device studies.

Staff at the community centers introduced the program to their members via Facebook Live (Meta Platforms, Inc.). Interested members were then contacted and screened by our trained research assistant through telephone calls. For those eligible to participate in the program, the research assistant provided a detailed explanation of the study, obtained consent, and collected baseline data at the community centers.

### Randomization

The participants were randomly assigned to either the intervention or control group using the Research Randomizer software (Social Psychology Network) to generate the group assignments. These assignments were sealed and opened sequentially by the principal investigator at the time of randomization. To achieve the highest possible degree of double-blinding, participants were informed only that the intervention aimed to promote the use of WMDs; they were not informed of their specific group assignment (intervention or control). In addition, the research assistant who collected the data was blinded to group allocation; however, the providers, including community center staff, were not.

### Intervention

#### Device Setup and Participant Instructions

After completing baseline data collection and before randomization, participants were provided with a package containing a WMD (ProVista Care; Provista Group), a prepaid SIM card, a blood pressure monitor, and a pulse oximeter. ProVista Care, a commercially available device, was selected as the WMD for this study due to its validation, affordability, and ability to perform various functions comparable to other WMDs. These functions include fall detection, location and activity tracking, blood pressure, pulse, and oxygen saturation monitoring, as well as medication and appointment reminders and telephone capabilities, enhancing the relevance of study findings for real-world implementation. The data collected from ProVista Care could be synchronized and transferred to the server after participants installed the ProVista Care app on their smartphones. The WMD was designed to be worn on the wrist and secured by an elastic band. All participants were encouraged to wear the WMD as frequently and for as long as possible throughout the study period.

The research team developed a computerized decision support model based on the guidelines of the American Heart Association, Hospital Authority, and Department of Health to guide an alert system [30]. When abnormal readings or signs, such as low oxygen levels, irregular pulse rhythm, or falls, are detected by the WMD, a health alert is sent to the smartphone of a nurse case manager for prompt assessment and management. The case manager followed an algorithm developed according to the guidelines from the National Institute for Health and Care Excellence to manage participants and, if necessary, refer them to a community center or a higher level of care, such as the emergency department. For instance, if a participant’s heart rate exceeded 100 and they experienced sweating and nausea, the case manager would refer them to the hospital. When deemed necessary, the nurse case manager would refer participants to social workers based on referral guidelines collaboratively developed by the nurse case managers and social workers. Examples of referral reasons include the need for homemaking services due to a high fall risk detected by the WMD, the need for transportation services for those frequently missing medical appointments due to mobility restrictions, and requests for information on fitness classes organized by the community center for participants identified as prehypertensive through daily WMD monitoring.

A 1-hour training session was provided to all participants via Zoom (Zoom Video Communications), covering how to use the WMD, blood pressure monitor, and pulse oximeter; create an account for the ProVista Care app; and synchronize data with their smartphones. Participants were required to pass a practical test demonstrating their ability to operate the devices before they could take the WMD from the community centers. The questions primarily focused on the steps for using the device. Participants were also provided with a telephone number for technical support, available during office hours from community center staff throughout the entire program period.

#### Intervention Group

Each participant in the intervention group received a 3-month Live With Wearable Monitoring Device program, delivered by CHWs under the supervision of a nurse case manager. Before the program began, 10 CHWs from 5 community centers of a nongovernmental organization, who expressed interest in the program, attended a 1-day training workshop. The training workshop was conducted by the project’s principal investigator and a nurse case manager, both with extensive experience in health promotion and older adults’ community care. The workshop consisted of 3 sessions: theoretical instruction, role-play, and testing. It combined theoretical knowledge with practical application, incorporating principles of health promotion and adult learning theories to equip CHWs with the competencies needed to effectively support older adults in the community. The first session concentrated on theoretical input, covering essential topics such as the use of the WMD and related apps, an introduction to the assessment tool (the Omaha System), and the application of intervention protocols and referral guidelines. Through interactive presentations and discussions, CHWs gained a comprehensive understanding of these concepts and their practical implications for delivering effective care.

The second session included role-playing activities, where CHWs engaged in simulated case scenarios to apply the knowledge and skills acquired in the theoretical session. This hands-on approach enabled CHWs to practice problem-solving, decision-making, and communication skills in a realistic context, with guidance and feedback from the facilitators.

Finally, all 10 CHWs underwent a competency assessment, consisting of a 10-item multiple-choice test, to ensure they were knowledgeable and skilled enough to provide care within the scope of the program. This assessment validated their learning outcomes and readiness to effectively undertake their roles as CHWs. The questions covered aspects such as understanding the WMD and related apps, familiarity with the Omaha System, application of intervention protocols, and practical implications for delivering effective care.

Participants in the intervention group received a home visit from a CHW and a nurse case manager during the first month, followed by biweekly telephone calls from the CHW from the third to the twelfth week. This arrangement has been validated as feasible for both participants and providers, demonstrating effectiveness in our previous studies [31,32].

The intervention components of this program explicitly focused on facilitating the 3 motivational factors outlined by SDT: autonomy, competence, and relatedness. During the first and only home visit, the CHWs and the nurse case manager visited participants at their homes to provide in-person demonstrations on how to use the devices correctly and to identify any environmental factors that might facilitate or hinder their use of the WMD. During this visit, the nurse case manager engaged participants in a collaborative discussion to explore the features of the WMD that each individual might find beneficial, utilizing the Omaha System. The Omaha System is a comprehensive assessment, intervention, and evaluation tool for community-based practice, covering 42 health and social problems across the domains of environmental, psychological, physiological, and health-related behaviors [33]. After discussions among the research team, the nurse case manager, and the social workers, 21 health and social problems were identified as relevant to, or potentially preventable by, the features of the WMD in this study. For instance, the medication reminder feature could assist participants struggling with medication adherence. The nurse case manager encouraged participants to explore and utilize the features of the WMD that were linked to their identified health and social problems, providing suggestions on usage duration and frequency, as well as instructions for integrating these features into their daily routines. Participants were empowered to adjust or modify their own schedules to ensure efficient and effective utilization of the WMD, with support from the nurse case manager and CHWs. Family members or primary caregivers were also encouraged to participate in discussions, providing feedback and support to enhance the sense of relatedness and social support. During the home visit, CHWs assisted the nurse case manager with health assessments and engaged in collaborative discussions with participants to formulate codeveloped goals and action plans. The CHWs supported, encouraged, and motivated participants to regularly use the WMD, fostering intrinsic motivation and ownership of their health management journey. Subsequent telephone calls were used for follow-ups, progress tracking, and discussions on goal achievement between the CHWs and participants. When necessary, goals and action plans were collaboratively modified with input from participants. Additionally, participants were encouraged to contact the CHWs with any problems or concerns related to the use of the WMD, promoting open communication and ongoing support.

#### Control Group

Similar to the intervention group participants, those in the control group could utilize the features available on the WMD. When abnormalities, such as high blood pressure, were detected, the nurse case manager followed the intervention protocol and called the participants for treatment and follow-up. However, the services provided were one-off, with no regular or continuous support from CHWs, the nurse case manager, or social workers.

### Outcome Measures

#### Overview

This study measured 3 dimensions of outcomes: usability of the WMD, feasibility, and effectiveness of the program. A description of each measure is presented in the following sections.

#### Usability of the WMD

Participants were required to complete a questionnaire assessing their attitudes toward using the WMD, perceived usefulness, perceived ease of use, self-efficacy in using the device, anxiety level, and facilitating conditions for using the device. The questionnaire was adapted from Chen and Chan [33]. This 10-point Likert scale ranges from 1 (strongly disagree) to 10 (strongly agree), with higher scores indicating a better outcome, except for the anxiety level, where higher scores reflect a greater level of anxiety associated with using the WMD. The questionnaire has been widely used and validated in prior empirical studies [34,35].

#### Feasibility of the Program

Feasibility refers to an assessment of the practicality of the study [36]. In this program, feasibility was operationalized through the evaluation of the recruitment rate, attrition rate, incidence of reported adverse events (eg, discomfort, injuries), and incidence of reported technical difficulties (eg, synchronizing data from the WMD to the smartphone app). The recruitment rate was calculated by dividing the number of participants who were recruited and randomized by the number of eligible participants. The attrition rate refers to the number of participants who withdraw or are lost to follow-up. Recruitment and attrition rates, as well as the incidence of reported adverse events and technical difficulties, were recorded by designated staff at each community center and by the nurse.

#### Effectiveness of the Program

Effectiveness outcomes included continued use intention, adherence rate, quality of life, self-efficacy, and health service utilization. Continued use intention, the primary subjective outcome of this study, was measured using a 3-item, 7-point Likert scale adapted from Bhattacherjee [36] and Windasari et al [28]. The 7-point scale ranges from 1 (strongly agree) to 5 (strongly disagree), with lower scores indicating a higher likelihood of continuing to use the WMD. This outcome was included to assess the sustained use of the device beyond the study period.

The adherence rate, the objective primary outcome, was measured by recording the number of days participants wore their device and the average time it was worn per day in our database. This information was automatically uploaded to the system whenever participants wore the device. Quality of life was assessed using the Hong Kong version of the EQ-5D-5L [37,38]. The EQ-5D-5L consists of 5 dimensions: mobility, self-care, usual activities, pain or discomfort, and anxiety or depression. The instrument has been widely used globally. Self-efficacy was measured using the Chinese version of the General Self-Efficacy Scale (CGSE) [39]. This scale uses a 4-point Likert format, with higher scores indicating greater levels of self-efficacy in using the WMD. The scale has been extensively validated and is recognized for its high reliability [32]. Health service utilization includes the number of visits to a general practitioner’s office, emergency department, hospital, and general outpatient clinic. Participants reported these data, which were then confirmed using medical and attendance certificates, demonstrating good reliability [31].

Background demographics, including age, gender, marital status, educational level, occupation, living status, primary caregiver, frequency of caretaking support, medical history, and eHealth literacy, were collected at baseline to account for potential group differences.

### Data Collection

Data collection was conducted at baseline (T0), 1 month (T1), 3 months (T2), and 6 months (T3), with T2 representing the immediate postintervention assessment and T3 measuring the sustained effects of the program. A trained research assistant, who was blinded to group allocation and not involved in either the intervention or control group, called the participants by telephone to collect the data.

### Statistical Analysis

Statistical tests were conducted using SPSS version 26 (IBM Corp.). Descriptive analyses were performed to summarize the baseline demographic data, which were presented as mean and SD for continuous variables, median and interquartile range for nonnormally distributed continuous variables, and percentage and frequency for categorical variables. The study used generalized estimating equations to assess differences or changes between the intervention and control groups (between-group effects), within-group (time) effects, and interaction effects (group × time). All outcomes were analyzed using a first-order autoregressive structure. Intention-to-treat analysis was implemented as the primary method for addressing missing data. A result was considered significant when the *P* value (level of significance) was less than 0.05 for a 2-tailed test.

### Sample Size

We conducted a power analysis to determine the sample size required for this study. Considering an α value of .05, a power of 80%, and an effect size of 0.72 derived from previous studies using the same primary outcome [40], we concluded that each group should comprise 32 participants. To accommodate a potential dropout rate of 10%, the total sample size for this study was set at 70, with 35 participants assigned to each group.

### Ethical Consideration

The ethical application was approved by the Human Ethics Subcommittee of the Hong Kong Polytechnic University (HSEARS20220429001) before the program’s commencement. The study is registered at ClinicalTrials.gov (NCT05269303). All eligible participants who joined the program signed a consent form. All documents, including the SPSS file, were encrypted, and only members of the research team, including the research assistant, had access to the password.

## Results

### Participant Flow

**Figure 2:**
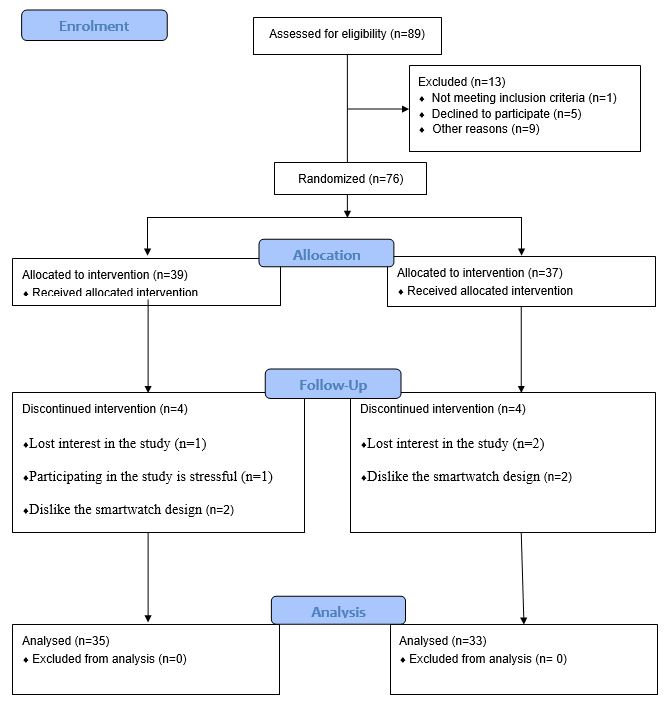
CONSORT (Consolidated Standards of Reporting Trials) flow diagram.

A total of 89 older adults expressed interest and were assessed for eligibility, leading to the recruitment of 76 participants from 5 community centers. They were randomly assigned to either the intervention group (n=39) or the control group (n=37). During the 6-month program, 4 participants from each group dropped out for various reasons, including loss of interest (n=3), dislike of the smartwatch design (n=4), and feeling pressure during the study (n=1; Figure 2 and Multimedia Appendix 1; see also [41]).

### Baseline Characteristics

Baseline demographic characteristics were balanced across the 2 groups (Table 1). Participants (N=76) had an average age of 74 (SD 7.6) years, with only 6 (8%) having no formal education. All but 1 participant were retired, and 30 (39%) lived alone. The majority (69/76, 91%) reported having adequate or more than adequate financial resources, primarily from the old age living allowance (28/76, 37%), family support (26/76, 34%), and personal savings (25/76, 33%). Hypertension (38/76, 50%) and chronic pain (38/76, 50%) were the most prevalent chronic diseases among the participants. Their eHealth literacy level was moderate, with an average score of 25.0 out of 40 on the Chinese version of the eHealth Literacy Scale [42].

**Table 1:** Demographic characteristics of the participants (N=76).

Demographic characteristics		Total (N=76)	Intervention group (n=39)	Control group (n=37)	*P* value
Gender, n (%)					.88
	Male	17 (22)	9 (23)	8 (22)	
	Female	59 (78)	30 (77)	29 (78)	
Age (years), mean (SD)		73.74 (7.62)	74.74 (8.16)	72.68 (6.95)	.36
Education level, n (%)					.30
	No formal education	6 (8)	2 (5)	4 (11)	
	Primary	31 (41)	18 (46)	13 (35)	
	Secondary	34 (45)	15 (38)	19 (51)	
	Tertiary or above	5 (7)	4 (10)	1 (3)	
Employment status, n (%)					>.99
	Part-time	1 (1)	1 (3)	0 (0)	
	Retired	75 (99)	38 (97)	37 (100)	
Living status, n (%)					.55
	Alone	30 (39)	14 (36)	16 (43)	
	With spouse	23 (30)	14 (36)	9 (24)	
	With family	23 (30)	11 (28)	12 (32)	
Financial status, n (%)					.91
	More than adequate	20 (26)	9 (23)	11 (30)	
	Adequate	49 (64)	26 (67)	23 (62)	
	Inadequate	5 (7)	3 (8)	2 (5)	
	Very inadequate	2 (3)	1 (3)	1 (3)	
Income, n (%)					
	Wage	1 (1)	1 (3)	0 (0)	>.99
	Family	26 (34)	15 (38)	11 (30)	.42
	Savings	25 (33)	14 (36)	11 (30)	.57
	Retirement pension	16 (21)	6 (15)	10 (27)	.21
	Comprehensive Social Security Assistance	7 (9)	5 (13)	2 (5)	.43
	Old age living allowance	28 (37)	16 (41)	12 (32)	.44
	Normal disability allowance	1 (1)	0 (0)	1 (3)	.49
	Higher disability allowance	2 (3)	0 (0)	2 (5)	.23
Comorbidity, n (%)					
	Chronic pain	38 (50)	17 (44)	21 (57)	.25
	Chronic obstructive pulmonary disease	5 (7)	3 (8)	2 (5)	>.99
	Hypertension	38 (50)	22 (56)	16 (43)	.25
	Diabetes	22 (29)	14 (36)	8 (22)	.17
	Genital disease	5 (7)	2 (5)	3 (8)	.67
	Stroke	3 (4)	2 (5)	1 (3)	>.99
	Cancer	8 (11)	4 (10)	4 (11)	>.99
	Arthritis	14 (18)	8 (21)	6 (16)	.63
	Depression	2 (3)	2 (5)	0 (0)	.49
	Cardiac disease	8 (11)	4 (10)	4 (11)	>.99
	Cataract	5 (7)	2 (5)	3 (8)	.67
eHealth literacy, mean (SD)		24.96 (7.34)	24.51 (7.71)	25.43 (7.01)	.82

### Usability of the WMD

**Figure 3:**
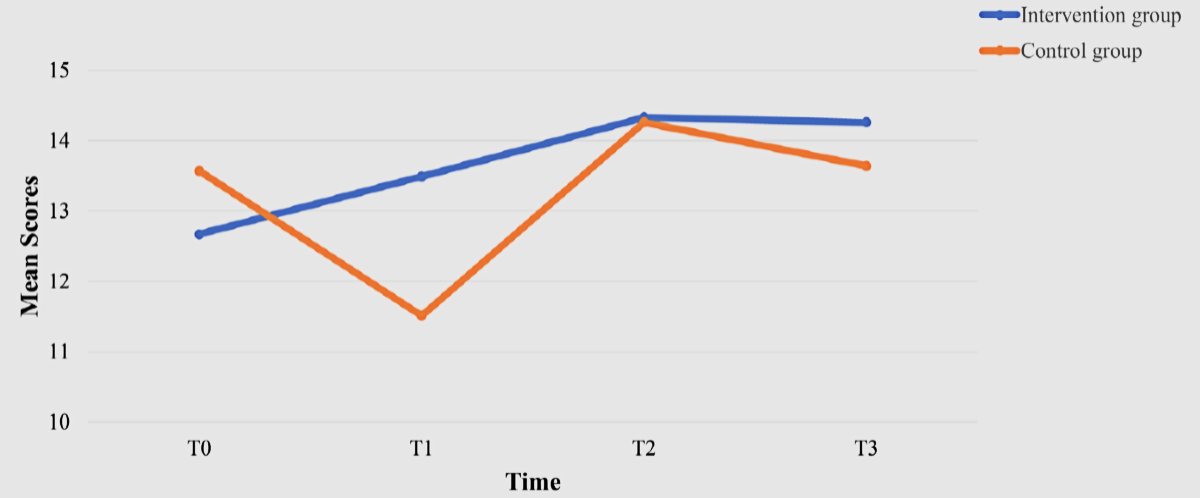
Perceived ease of use scores of the 2 groups over time.

**Figure 4:**
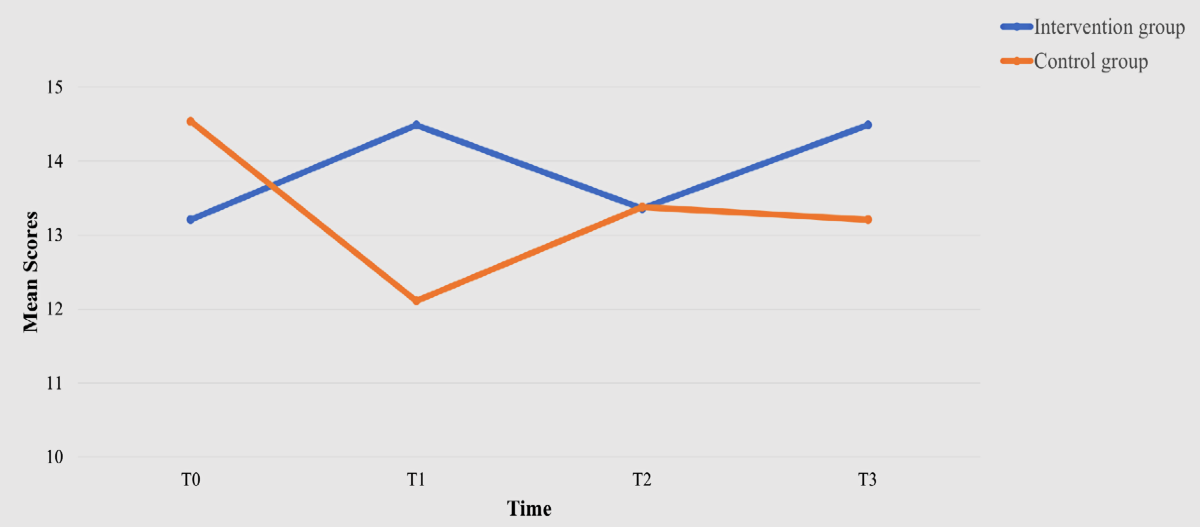
Self-efficacy scores for device use in the 2 groups over time.

**Figure 5:**
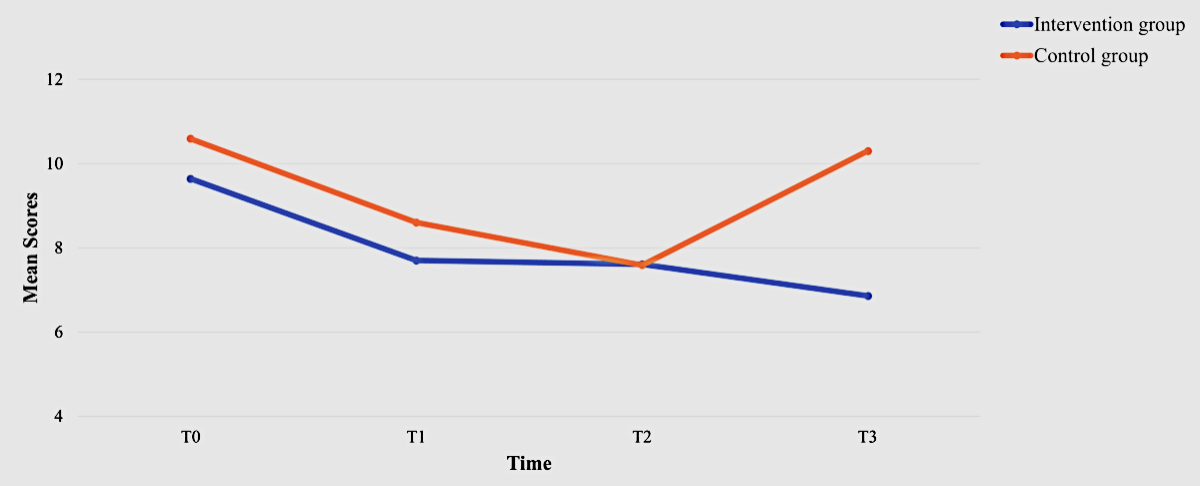
Anxiety scores of the 2 groups over time.

Although the mean values of attitudes toward using the WMD in the intervention group were higher than those in the control group at all 4 time points (Table 2), the between-group differences were not statistically significant (*P*=.42). A significant within-group effect was observed at T2 (β=–1.82, 95% CI –3.21 to –0.43, *P*=.01; Table 3). Similarly, while no statistically significant (*P*=.40) between-group differences were found at any of the 4 time points regarding perceived usefulness, significant within-group effects were demonstrated at T1 (β=–4.70, 95% CI –7.51 to –1.89, *P*=.001) and T2 (β=–4.42, 95% CI –6.98 to –1.85, *P*=.001; Table 3). The mean values of perceived ease of use improved from T0 to T3 in the intervention group (Figure 3). A significant within-group effect was observed at T1 (β=–2.05, 95% CI –3.85 to –0.26, *P*=.02), along with an interaction effect on perceived ease of use (β=2.87, 95% CI 0.56-5.18, *P*=.01) for the intervention group (Table 3). The intervention group recorded the highest mean self-efficacy scores at T3, while the control group achieved its highest mean scores at T0 (Table 2). The Mann-Whitney *U* test indicated that the intervention group had a significantly greater self-efficacy level than the control group at T1 (*z*=–2.05, *P*=.002; Figure 4). Additionally, there was a significant within-group effect on self-efficacy at T1 (β=–2.43, 95% CI –3.78 to –1.07, *P*<.001) and significant interaction effects at T1 (β=3.71, 95% CI 1.76-5.66, *P*<.001) and T3 (β=2.61, 95% CI 0.22-5.00, *P*=.03) for the intervention group (Table 2). The intervention group demonstrated a statistically significant lower anxiety level associated with using the WMD at T3 compared with the control group (*z*=2.10, *P*=.003). The mean anxiety levels in the intervention group also decreased from T0 to T3 (Figure 5). Significant within-group differences in anxiety levels were observed at T1 (β=–2.00, 95% CI –3.67 to –0.32, *P*=.02) and T2 (β=–3.01, 95% CI –4.74 to –1.28, *P*<.001; Table 3). The mean values for facilitating conditions related to the use of the WMD improved in the intervention group; however, no statistically significant (*P*=.16) between-group effect was observed. Significant within-group effects on facilitating conditions were identified at T1 (β=–4.47, 95% CI –7.58 to –1.36, *P*=.005), along with interaction effects at T1 (β=5.44, 95% CI 1.04-9.85, *P*=.01) for the intervention group (Table 3).

**Table 2:** Mean values of the usability and effectiveness outcomes for the 2 groups at different time points.

Outcomes and groups				Mean		SE		95% Wald CI
Attitude toward using WMD^a^								
	Control group							
		T3	13.70		0.66		12.39-15.00	
		T2	12.21		0.77		10.69-13.72	
		T1	12.63		0.76		11.13-14.12	
		T0	14.03		0.55		12.94-15.11	
	Intervention group							
		T3	14.09		0.76		12.60-15.57	
		T2	13.81		0.77		12.30-15.31	
		T1	14.19		0.68		12.85-15.53	
		T0	14.64		0.53		13.61-15.67	
Perceived usefulness								
	Control group							
		T3	19.73		1.06		17.64-21.81	
		T2	17.85		1.13		15.64-20.07	
		T1	17.57		1.29		15.04-20.10	
		T0	22.27		0.89		20.53-24.01	
	Intervention group							
		T3	19.97		1.29		17.44-22.50	
		T2	19.67		1.35		17.03-22.31	
		T1	18.97		1.13		16.75-21.19	
		T0	21.28		0.77		19.78-22.78	
Perceived ease of use								
	Control group							
		T3	13.64		0.73		12.21-15.07	
		T2	14.26		0.64		13.00-15.53	
		T1	11.51		0.88		9.79-13.24	
		T0	13.57		0.50		12.59-14.54	
	Intervention group							
		T3	14.26		0.89		12.50-16.01	
		T2	14.33		0.78		12.80-15.86	
		T1	13.49		0.69		12.14-14.83	
		T0	12.67		0.64		11.41-13.92	
Anxiety								
	Control group							
		T3	10.30		0.85		8.64-11.96	
		T2	7.59		0.80		6.02-9.16	
		T1	8.60		0.69		7.25-9.95	
		T0	10.59		0.62		9.38-11.81	
	Intervention group							
		T3	6.86		0.76		5.37-8.34	
		T2	7.61		0.70		6.24-8.98	
		T1	7.70		0.66		6.41-8.99	
		T0	9.64		0.66		8.34-10.94	
Self-efficacy of using the device								
	Control group							
		T3	13.21		0.71		11.83-14.59	
		T2	13.38		0.56		12.29-14.48	
		T1	12.11		0.61		10.91-13.31	
		T0	14.54		0.54		13.48-15.60	
	Intervention group							
		T3	14.49		0.81		12.90-16.07	
		T2	13.36		0.71		11.97-14.75	
		T1	14.49		0.60		13.31-15.66	
		T0	13.21		0.61		12.00-14.41	
Facilitating conditions								
	Control group							
		T3	32.97		1.49		30.05-35.89	
		T2	31.85		1.41		29.10-34.61	
		T1	29.77		1.49		26.84-32.70	
		T0	34.24		1.32		31.65-36.83	
	Intervention group							
		T3	32.71		1.82		29.15-36.28	
		T2	32.53		1.72		29.16-35.90	
		T1	32.46		1.44		29.64-35.28	
		T0	31.49		1.46		28.62-34.35	
Continued use intention								
	Control group							
		T3	15.18		0.70		13.80-16.56	
		T2	13.71		0.90		11.95-15.46	
		T1	13.31		0.92		11.52-15.11	
	Intervention group							
		T3	16.23		0.79		14.69-17.77	
		T2	14.44		0.92		12.64-16.25	
		T1	14.32		0.74		12.87-15.78	
Adherence rate (number of days/week)								
	Control group							
		T3	3.24		0.498		2.27-4.22	
		T2	3.91		0.480		2.97-4.85	
		T1	5.40		0.366		4.68-6.12	
	Intervention group							
		T3	4.86		0.495		3.89-5.83	
		T2	6.25		0.292		5.68-6.82	
		T1	6.38		0.266		5.86-6.90	
Adherence rate (number of hours/day)								
	Control group							
		T3	4.48		0.74		3.03-5.94	
		T2	5.06		0.79		3.52-6.60	
		T1	6.67		0.92		4.87-8.48	
	Intervention group							
		T3	5.79		0.88		4.06-7.51	
		T2	9.29		0.82		7.68-10.90	
		T1	9.68		0.96		7.80-11.55	
EQ-5D-5L index value								
	Control group							
		T3	0.74		0.03		0.69-0.79	
		T2	0.75		0.02		0.71-0.79	
		T1	0.72		0.01		0.69-0.75	
		T0	0.72		0.02		0.69-0.75	
	Intervention group							
		T3	0.79		0.03		0.74-0.84	
		T2	0.79		0.02		0.75-0.83	
		T1	0.78		0.02		0.74-0.82	
		T0	0.75		0.03		0.69-0.81	
EQ-5D-5L visual analog scale								
	Control group							
		T3	75.00		2.033		71.02-78.98	
		T2	72.79		2.213		68.46-77.13	
		T1	72.17		2.699		66.88-77.46	
		T0	68.51		2.563		63.49-73.54	
	Intervention group							
		T3	76.86		2.921		71.13-82.58	
		T2	74.86		2.668		69.63-80.09	
		T1	72.27		3.016		66.36-78.18	
		T0	73.59		2.384		68.92-78.26	
The Chinese version of the General Self-Efficacy Scale								
	Control group							
		T3	24.70		1.19		22.36-27.03	
		T2	24.74		1.07		22.63-26.84	
		T1	23.94		0.99		22.00-25.89	
		T0	24.51		1.04		22.48-26.55	
	Intervention group							
		T3	28.71		0.91		26.93-30.50	
		T2	27.44		1.06		25.38-29.51	
		T1	26.30		1.02		24.30-28.30	
		T0	27.82		1.09		25.69-29.95	
Health service utilization								
	Control group							
		T3	0.48		0.15		0.19-0.78	
		T2	0.68		0.19		0.30-1.06	
		T1	0.51		0.16		0.20-0.82	
		T0	0.57		0.24		0.10-1.04	
	Intervention group							
		T3	0.57		0.19		0.19-0.95	
		T2	0.53		0.19		0.16-0.89	
		T1	0.86		0.26		0.36-1.37	
		T0	1.10		0.36		0.40-1.81	

^a^WMD: wearable monitoring device.

**Table 3:** Parameter estimates of outcomes.

Parameter estimates		β	SE	95% CI	Wald *χ*^2^ (*df*)	*P* value^a^
Attitude toward using WMD^b^						
	Intercept	14.027	0.5532	12.943 to 15.111	642.908 (1)	*<.001*
	Intervention group	0.614	0.7643	–0.884 to 2.112	0.645 (1)	.44
	Time=3	–0.330	0.7988	–1.896 to 1.236	0.171 (1)	.68
	Time=2	–1.821	0.7104	–3.213 to –0.429	6.572 (1)	*.01*
	Time=1	–1.398	0.9149	–3.192 to 0.395	2.336 (1)	.13
	Intervention group × time=3	–0.225	1.1350	–2.450 to 1.999	0.039 (1)	.84
	Intervention group × time=2	0.986	1.0725	–1.116 to 3.088	0.845 (1)	.36
	Intervention group × time=1	0.947	1.1654	–1.338 to 3.231	0.660 (1)	.42
Perceived usefulness						
	Intercept	22.270	0.8879	20.530 to 24.011	629.091 (1)	*<.001*
	Intervention group	–0.988	1.1725	–3.286 to 1.310	0.710 (1)	.40
	Time=3	–2.543	1.4385	–5.362 to 0.276	3.125 (1)	.08
	Time=2	–4.417	1.3092	–6.983 to –1.851	11.384 (1)	*<.001*
	Time=1	–4.699	1.4354	–7.512 to –1.885	10.716 (1)	*.001*
	Intervention group × time=3	1.232	2.1029	–2.889 to 5.354	0.343 (1)	.56
	Intervention group × time=2	2.802	1.8610	–0.845 to 6.449	2.267 (1)	.13
	Intervention group × time=1	2.390	1.8589	–1.254 to 6.033	1.653 (1)	.20
Ease of use						
	Intercept	13.568	0.4977	12.592 to 14.543	743.197 (1)	*<.001*
	Intervention group	–0.901	0.8114	–2.491 to 0.689	1.233 (1)	.27
	Time=3	0.069	0.7383	–1.378 to 1.516	0.009 (1)	.93
	Time=2	0.697	0.6013	–0.481 to 1.876	1.344 (1)	.25
	Time=1	–2.053	0.9168	–3.850 to –0.256	5.016 (1)	*.02*
	Intervention group × time=3	1.522	1.1784	–0.788 to 3.831	1.667 (1)	.20
	Intervention group × time=2	0.970	0.9902	–0.971 to 2.910	0.959 (1)	.33
	Intervention group × time=1	2.873	1.1789	0.563 to 5.184	5.940 (1)	*.01*
Self-efficacy of using the device						
	Intercept	14.541	0.5386	13.485 to 15.596	728.731 (1)	*<.001*
	Intervention group	–1.335	0.8164	–2.935 to 0.265	2.676 (1)	.10
	Time=3	–1.328	0.8231	–2.942 to 0.285	2.605 (1)	.10
	Time=2	–1.158	0.7177	–2.565 to 0.249	2.604 (1)	.10
	Time=1	–2.426	0.6930	–3.784 to –1.068	12.259 (1)	*<.001*
	Intervention group × time=3	2.609	1.2178	0.222 to 4.996	4.590 (1)	*.03*
	Intervention group × time=2	1.314	1.0201	–0.685 to 3.313	1.660 (1)	.20
	Intervention group × time=1	3.708	0.9949	1.758 to 5.658	13.888 (1)	*<.001*
Anxiety						
	Intercept	10.595	0.6204	9.379 to 11.811	291.627 (1)	*<.001*
	Intervention group	–0.954	0.9089	–2.735 to 0.828	1.101 (1)	.29
	Time=3	–0.292	0.9682	–2.189 to 1.606	0.091 (1)	.76
	Time=2	–3.006	0.8830	–4.737 to –1.276	11.592 (1)	*<.001*
	Time=1	–1.995	0.8555	–3.671 to –0.318	5.436 (1)	*.02*
	Intervention group × time=3	–2.492	1.3337	–5.106 to 0.122	3.492 (1)	.06
	Intervention group × time=2	0.976	1.3199	–1.611 to 3.563	0.547 (1)	.46
	Intervention group × time=1	0.056	1.2547	–2.403 to 2.515	0.002 (1)	.96
Facilitating conditions						
	Intercept	34.243	1.3215	31.653 to 36.833	671.441 (1)	*<.001*
	Intervention group	–2.756	1.9695	–6.616 to 1.104	1.958 (1)	.16
	Time=3	–1.274	1.6801	–4.566 to 2.019	0.575 (1)	.45
	Time=2	–2.390	1.5979	–5.522 to 0.742	2.238 (1)	.13
	Time=1	–4.472	1.5865	–7.581 to –1.362	7.945 (1)	*.005*
	Intervention group × time=3	2.501	2.6279	–2.650 to 7.651	0.905 (1)	.34
	Intervention group × time=2	3.431	2.4679	–1.406 to 8.268	1.933 (1)	.16
	Intervention group × time=1	5.444	2.2479	1.038 to 9.850	5.865 (1)	*.01*
Continued use intention						
	Intercept	13.314	0.9168	11.517 to 15.111	210.925 (1)	*<.001*
	Intervention group	1.010	1.1789	–1.301 to 3.321	0.734 (1)	.39
	Time=3	1.868	0.9286	0.048 to 3.687	4.045 (1)	*.04*
	Time=2	0.392	0.7277	–1.035 to 1.818	0.290 (1)	.59
	Intervention group × time=3	0.037	1.2013	–2.318 to 2.391	0.001 (1)	.98
	Intervention group × time=2	–0.271	0.9793	–2.191 to 1.648	0.077 (1)	.78
Adherence rate (number of days/week)						
	Intercept	5.400	0.3663	4.682 to 6.118	217.281 (1)	*<.001*
	Intervention group	0.978	0.4526	0.091 to 1.865	4.673 (1)	*.03*
	Time=3	–2.158	0.5265	–3.190 to –1.126	16.790 (1)	*<.001*
	Time=2	–1.488	0.4353	–2.341 to –0.635	11.690 (1)	*<.001*
	Intervention group × time=3	0.636	0.7397	–0.813 to 2.086	0.740 (1)	.39
	Intervention group × time=2	1.360	0.5964	0.191 to 2.529	5.199 (1)	*.02*
Adherence rate (number of hours/day)						
	Intercept	6.671	0.9205	4.867 to 8.476	52.528 (1)	*<.001*
	Intervention group	3.004	1.3286	0.400 to 5.608	5.113 (1)	*.02*
	Time=3	–2.187	0.9280	–4.005 to –0.368	5.552 (1)	*.02*
	Time=2	–1.613	0.7805	–3.142 to –0.083	4.269 (1)	*.04*
	Intervention group × time=3	–1.703	1.4599	–4.565 to 1.158	1.361 (1)	.24
	Intervention group × time=2	1.229	1.0430	–0.816 to 3.273	1.388 (1)	.24
EQ-5D-5L index value						
	Intercept	0.719	0.0162	0.687 to 0.751	1959.643 (1)	*<.001*
	Intervention group	0.029	0.0345	–0.038 to 0.097	0.715 (1)	.40
	Time=3	0.018	0.0190	–0.019 to 0.055	0.898 (1)	.34
	Time=2	0.029	0.0192	–0.008 to 0.067	2.316 (1)	.13
	Time=1	0.004	0.0121	–0.020 to 0.027	0.098 (1)	.75
	Intervention group × time=3	0.028	0.0354	–0.042 to 0.097	0.605 (1)	.44
	Intervention group × time=2	0.014	0.0338	–0.052 to 0.080	0.175 (1)	.68
	Intervention group × time=1	0.028	0.0289	–0.028 to 0.085	0.972 (1)	.32
EQ-5D-5L visual analog scale						
	Intercept	68.514	2.5630	63.490 to 73.537	714.561 (1)	*<.001*
	Intervention group	5.076	3.5007	–1.785 to 11.937	2.103 (1)	.15
	Time=3	6.486	2.5302	1.527 to 11.445	6.572 (1)	*.01*
	Time=2	4.281	2.0782	0.207 to 8.354	4.243 (1)	*.04*
	Time=1	3.658	3.2388	–2.690 to 10.006	1.276 (1)	.26
	Intervention group × time=3	–3.219	3.8121	–10.691 to 4.253	0.713 (1)	.40
	Intervention group × time=2	–3.009	3.0391	–8.966 to 2.947	0.980 (1)	.32
	Intervention group × time=1	–4.977	3.7929	–12.411 to 2.457	1.722 (1)	.19
The Chinese version of the General Self-Efficacy Scale						
	Intercept	24.514	1.0395	22.476 to 26.551	556.128 (1)	*<.001*
	Intervention group	3.307	1.5033	0.361 to 6.253	4.839 (1)	*.03*
	Time=3	0.183	1.0463	–1.867 to 2.234	0.031 (1)	.86
	Time=2	0.222	0.9018	–1.546 to 1.989	0.060 (1)	.81
	Time=1	–0.571	0.9197	–2.373 to 1.232	0.385 (1)	.53
	Intervention group × time=3	0.710	1.5155	–2.260 to 3.681	0.220 (1)	.64
	Intervention group × time=2	–0.598	1.4507	–3.441 to 2.245	0.170 (1)	.68
	Intervention group × time=1	–0.953	1.3783	–3.654 to 1.749	0.478 (1)	.49
Health service utilization						
	Intercept	0.568	0.2403	0.097 to 1.039	5.577 (1)	*.02*
	Intervention group	0.535	0.4317	–0.311 to 1.381	1.536 (1)	.21
	Time=3	–0.083	0.2838	–0.639 to 0.473	0.085 (1)	.77
	Time=2	0.109	0.2282	–0.338 to 0.556	0.228 (1)	.63
	Time=1	–0.053	0.2369	–0.518 to 0.411	0.051 (1)	.82
	Intervention group × time=3	–0.448	0.4665	–1.363 to 0.466	0.924 (1)	.34
	Intervention group × time=2	–0.684	0.4027	–1.473 to 0.106	2.882 (1)	.09
	Intervention group × time=1	–0.184	0.4097	–0.987 to 0.619	0.203 (1)	.65

^a^Italicized values are significant at *P*<.05.

^b^WMD: wearable monitoring device.

### Feasibility of the Program

The recruitment rate for this program was high, with only 13 potential participants (N=89, 15%) deemed ineligible. Reasons for ineligibility included not meeting the inclusion criteria (n=1), declining to participate (n=5), and citing other reasons such as eczema or unavailability (n=7). The attrition rate was low, with only 11% (8/76) of participants withdrawing from the study. Throughout the 6-month program, only 1 instance of a skin problem was reported. The 3 most frequently reported technical problems by participants were smartwatch malfunctions (15/96, 16%), inability to upload vital signs to the mobile app (13/96, 14%), and pace counter malfunctions (10/96, 10%).

### Effectiveness of the Program

**Figure 6:**
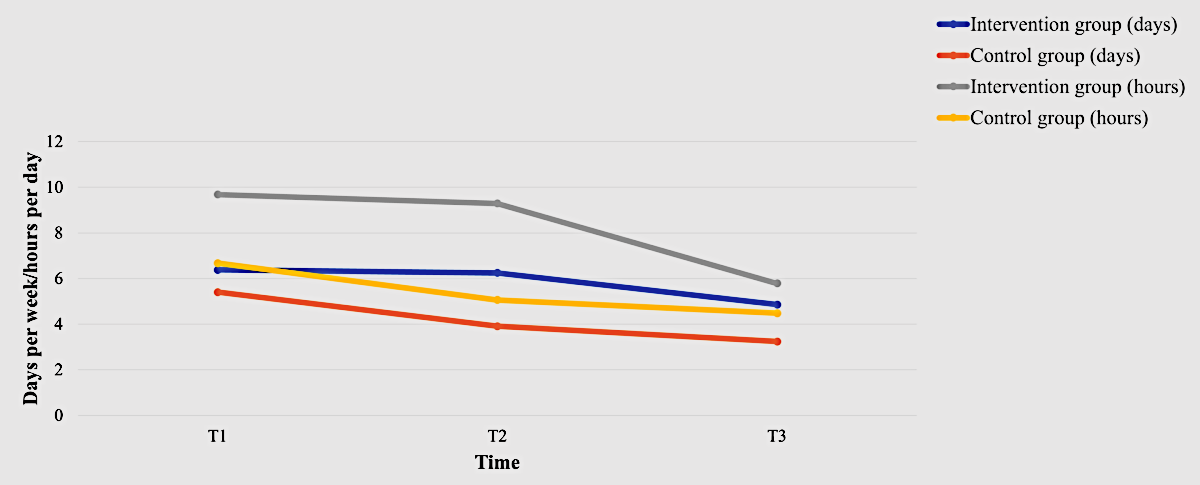
Adherence rate of the 2 groups over time.

**Figure 7:**
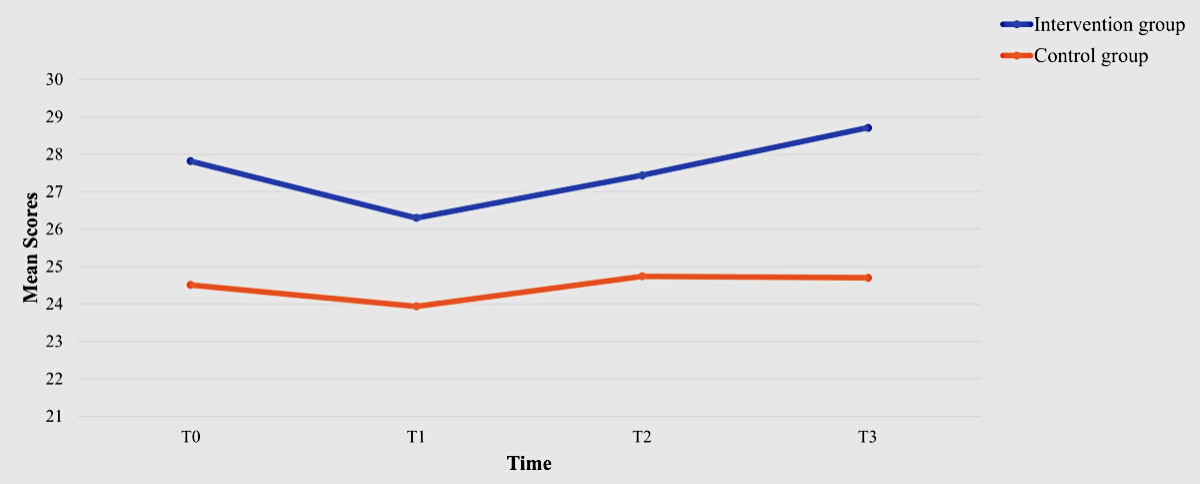
The Chinese version of the General Self-Efficacy Scale (CGSE) scores of the 2 groups over time.

Both groups showed an improvement in continued intention to use the WMD from T1 to T3 (Table 2), resulting in no statistically significant (*P*=.39) between-group differences. However, a within-group effect was observed at T3 (β=1.87, 95% CI of β 0.05-3.69, *P*=.04; Table 3). Regarding adherence rates, the intervention group demonstrated more frequent use of the smartwatch in terms of both days and hours. Statistically significant between-group differences were noted at all time points: T1 (*P*=.01), T2 (*P*<.001), and T3 (*P*=.02; Figure 6). Although the mean EQ-5D-5L index value of the intervention group improved from T0 to T3 and was higher than that of the control group at each time point (Table 2), no statistically significant between-group (*P*=.40), within-group (*P*=.75 for T1-T2, *P*=.12 for T1-T3, and *P*=.34 for T1-T4), or interaction effects (*P*=.32 for intervention × time=2; *P*=.68 for intervention × time=3; *P*=.44 for intervention × time=4) were observed. However, within-group effects on the EQ-5D-5L visual analog scale were noted at T2 (β=4.28, 95% CI of β 0.21-8.35, *P*=.04) and T3 (β=6.49, 95% CI of β 1.53-11.45, *P*=.01; Table 3). A statistically significant between-group effect was observed in self-efficacy, with the intervention group demonstrating a higher CGSE score (β=3.31, 95% CI of β 0.36-6.25, *P*=.03; Figure 7). However, no significant between-group (*P*=.21), within-group (*P*=.82 for T1-T2, *P*=.63 for T1-T3, and *P*=.77 for T1-T4), or interaction effects (*P*=.65 for intervention × time=2; *P*=.09 for intervention × time=3; *P*=.34 for intervention × time=4) were found regarding health service utilization.

## Discussion

### Principal Findings and Comparison With Previous Work

WMDs are increasingly recognized as valuable tools for ongoing health monitoring and the remote delivery of health care services. Despite their potential to support self-management across diverse populations, concerns about their sustained use persist. Therefore, this novel study investigates whether ongoing support from CHWs and other health care providers can enhance the usability, feasibility, and effectiveness of WMDs among community-dwelling older adults. Over a 6-month period, the study findings indicate that participants in the intervention group exhibited better attitudes toward the WMD, improved perceived ease of use, higher self-efficacy, and lower anxiety levels compared with the control group. Despite these positive outcomes, it was noted that the intention to continue using the WMD improved in both groups, with participants in the intervention group using the device more frequently. Additionally, while the quality-of-life index improved in the intervention group, the changes were not statistically significant, with only between-group effects observed regarding self-efficacy. Nonsignificant findings were noted for health service utilization across both groups. These results suggest that supplementary professional support may enhance certain outcomes related to the use of WMDs among older adults. Therefore, maintaining long-term access to such support could be essential for ensuring the ongoing use of WMDs.

Usability often reflects the ease and accessibility with which a user can interact with an app or device, serving as a significant determinant for the adoption of WMDs [43]. In this study, participants in the intervention group exhibited generally higher scores in attitude, perceived ease of use, and self-efficacy compared with those in the control group. Additionally, participants in the intervention group reported lower anxiety scores compared with those in the control group. These findings suggest that the ongoing support provided by CHWs may have empowered participants in the intervention group to develop the skills necessary for effectively navigating the WMDs and the monitoring system. This support likely contributed to achieving competency, as outlined in SDT [44]. In fact, the sustained support provided an ongoing learning opportunity for older adults as they engaged with a new device. This support may have facilitated their transition from novices to more proficient users, enhancing their ability to navigate the system and troubleshoot when necessary. This notion reflects an increase in autonomy, aligning with the principles of SDT [45]. Recent studies investigating the factors influencing the use of activity-tracking wearable devices among older adults have suggested that social influence plays a critical role within their social context [46,47]. Moreover, as participants received support and education to use the wearable devices, they began to view them more positively. This shift occurred as they not only learned to navigate the system but also experienced the devices’ benefits, integrating them into their daily lives [46,48]. Yildirim and Ali-Eldin [48] observed that the perceived usefulness of a wearable device is the strongest motivator for its continued use. Over time, participants’ attitudes and perceptions toward these devices evolved as their ease of use gradually increased [49,50]. This insight emphasizes the importance of providing ongoing support to sustain the use of wearable devices, especially during the initial stages of implementation. As noted in several existing studies, limited engagement during this early phase can lead to frustration and, ultimately, the abandonment of the devices altogether [48,50].

In terms of the intervention’s effectiveness, our study found that the intention to continue using the WMDs improved from T1 to T3 in both groups. While this is a positive outcome, it is important to note that intention to use may not always translate into actual usage [51]. This distinction is particularly relevant in this study, as although both groups showed an increased intention to use the WMDs, participants in the intervention group utilized the smartwatches more frequently than those in the control group. With the continued presence of social influence from CHWs, older adults in the intervention group may have been motivated to persist in using the WMDs [43]. This underscores the essential need for ongoing education to support older adults in their use of wearable devices. Therefore, it is insufficient to merely provide access to WMDs; ongoing support is necessary to ensure their effective utilization.

Despite the comprehensive nature of the intervention, which included ongoing support, no statistically significant differences were found concerning quality of life and health service utilization. Nonetheless, an improvement in the quality-of-life index value was observed. Additionally, only between-group effects were noted for self-efficacy, with the intervention group demonstrating higher CGSE scores. Undoubtedly, digital-based monitoring and cost-efficient smart wearable technologies for physical activity tracking represent an innovative platform for health promotion, allowing for the proactive identification and resolution of emerging biopsychosocial issues [52]. These technologies also facilitate the promotion of a healthy lifestyle among end users. In this study, the use of the Omaha System proved particularly beneficial for identifying, managing, and evaluating biopsychosocial and environmental problems, thereby promoting comprehensive health. Thus, the nonsignificant findings regarding quality of life present an intriguing aspect of the study. The inclusion of older adults with varying underlying chronic illnesses may warrant further consideration. As indicated in the “Results” section, many participants were dealing with chronic conditions such as pain, hypertension, diabetes, and arthritis, which can significantly affect quality of life. Factors contributing to a diminished quality of life often include a reduced ability to engage in practical and social activities, with chronic pain being a notable influence [53]. This highlights the possibility that some participants may require a higher dose or intensity of the intervention than others, warranting further investigation. Future trials should consider adjusting the intervention dose to meet the unique needs of participants, ensuring a personalized approach to implementing WMDs. The statistically nonsignificant findings regarding health service utilization may indicate comparable levels of utilization across both the intervention and control groups. This could be attributed to the WMD’s design, which includes a monitoring platform intended to detect and respond to health-related issues.

The feasibility outcomes, characterized by high recruitment, retention, and low attrition rates, are noteworthy in this study, especially given the 6-month duration. While it remains unclear what happens to WMD usage after the intervention concludes, it can be inferred that most participants utilized the WMD throughout the entire study period. Many individuals tend to abandon WMDs shortly after starting to use them, highlighting the significance of this finding [54,55]. In fact, most users tend to abandon WMDs within 3-6 months following their purchase [55-57]. In this study, participants in both the intervention and control groups received the same initial support; however, those in the intervention group benefited from ongoing assistance. Moreover, any abnormalities noted in the monitoring records of participants in the control group were actively followed up by the nurse case manager, which may have fostered a social effect by reassuring participants that support was available. This social effect and initial engagement may have stimulated further interest in the WMD and its potential benefits, thereby sustaining participants’ interest regardless of their assigned group [43]. Despite these assertions, a qualitative exploration may be warranted to capture participants’ experiences and gain an in-depth understanding of why they continue to use the WMD. Such a study could complement the findings from this trial, shedding light on the factors that motivate older adults to adopt and maintain their use of WMDs.

### Limitations

Although the study provides valuable insights into the utilization of WMDs among older adults, several limitations warrant attention. First, the study utilized a specific WMD designed for ongoing monitoring. Given the diverse range of WMDs available on the market, exploring other types of devices may yield different outcomes worth investigating further. Second, while the intention to continue using the app was assessed, it remains uncertain whether participants will actually use the WMD beyond the 6-month study period. Future studies should consider extending the duration of research to better understand the long-term actual usage of WMDs among older adults. Applying an implementation science approach grounded in a critical realist perspective may help uncover the generative mechanisms that explain the sustained usage of WMDs in this population. Additionally, the study did not involve older adults in the development process, which may limit how well the intervention aligns with their perspectives and preferences.

### Conclusion

WMDs have the potential to support self-management among community-dwelling older adults; however, they may not be sufficient on their own, necessitating ongoing support from health care professionals. This ongoing support is essential for building competence and autonomy, as well as fostering relatedness among older adults, in accordance with SDT. Future studies should consider customizing the dose or intensity of the intervention to meet the individual needs of patients, rather than adopting a one-size-fits-all approach.

## Appendix

Multimedia Appendix 1 CONSORT (Consolidated Standards of Reporting Trials) checklist.

## Abbreviations

CGSE: the Chinese version of the General Self-Efficacy Scale
CHW: community health worker
LWMD: Live With Wearable Monitoring Device
SDT: Self-Determination Theory
WMD: Wearable Monitoring Device

## Data Availability

The data presented in this study are available upon reasonable request from the corresponding author.
